# Employees and the National Insurance Act

**Published:** 1912-06-01

**Authors:** 


### NATIONAL INSURANCE ACT DEPARTMENT.

### EMPLOYEES AND THE NATIONAL INSURANCE ACT.

### XIII.—The Insurance of Hospital Nurses—(*continued*).

This week we continue the consideration of the insurance of the female nursing staffs of voluntary hospitals, dealing more particularly with the benefits receivable. The standard benefits are five in number and are described in the Act as (*a*) Medical benefit, (*b*) Sanatorium benefit, (*c*) Sickness benefit, (*d*) Disablement benefit, and (*e*) Maternity benefit. Medical benefit does not apply to insured persons in Ireland, but apart from this all nurses becoming insured at the ordinary scales of contribution will be entitled to all the benefits enumerated on the fulfilment of the conditions under which they become payable. Medical benefit includes medical treatment and medicine for the insured nurse (not for her family or dependants) and certain kinds of surgical appliances. The nurse becoming insured will be at liberty to choose the doctor whom she wishes to attend her, provided his name is included in the list of doctors published by the local Insurance Committee, and that he is willing to attend her. Medical benefit does not come into operation until six months after the commencement of the Act—that is to say, the earliest date on which it will be possible for a nurse to receive free medical attendance, under the Act, will be January 15, 1913. We indicated last week that in many cases this benefit will be superfluous, so far as hospital nurses are concerned, by reason of the fact that they are already cared for by the hospitals employing them. This was fully realised when the Bill was in Committee, and a very important amendment of the original scheme, known as the Harmsworth amendment, was incorporated in the Act to meet this special case.

#### When Medical Benefit is Already Provided.

The purport of this amendment is that in the case of persons who are entitled to receive medical attendance and treatment under any system or through any institution existing at the time of the passing of the Act (December 16, 1911), which is duly approved by the Insurance Committee and the Insurance Commissioners, such medical attendance and treatment may be treated as, or as part of, their medical benefit as provided under the Act. The amendment further provides that where such an arrangement is made, the Insurance Committee may be required to contribute, towards the expenses, the whole or any part of the sums with which the Committee would have been charged had no such arrangement been made, provided that no person affected by such scheme shall be deprived of the right of being attended by the doctor chosen in accordance with the regulations to which we have already referred.

It would thus appear to be possible for the hospital authorities to enter into arrangements with the local Insurance Committees for the continuation of the provision of medical treatment to their nursing staffs as part of the statutory benefits under the Act, and may receive in consideration some part, at least, of the expense incurred.

On the recommendation of the Insurance Committee, a nurse who is insured and is suffering from consumption is given free treatment in a sanatorium or other institution, or in such other way as may be suitable for the case.

#### Sickness Benefit for Nurses.

The ordinary rate of sickness benefit obtainable by female nurses is 7s. 6d. a week. These payments may be continued for twenty-six weeks, or for such shorter period as the nurse is incapable of work through sickness or other bodily or mental disablement. It must be noted, however, that no payment will accrue until the fourth day of illness and that illnesses occurring at separate periods are all counted as one illness for the purpose of reckoning the twenty-six weeks, unless a period of twelve months elapses and fifty weekly contributions are paid between the end of the one illness and the beginning of the next. To become entitled to this benefit the nurse must have been insured for at least twenty-six weeks and have paid at least twenty-six contributions. It is not payable after the age of seventy. In the case of a nurse who is under twenty-one years of age and unmarried, the benefit will be at the reduced rate of 5s. a week for the first thirteen weeks, and the sum of 4s. a week for the second thirteen weeks. Reduction in the rate of benefit according to scale will also take place when the contributions are in arrear. Last week we pointed out that under Section 47 of the Act it may be possible for the hospital authorities to guarantee the payment of full remuneration to their nurses during the first six weeks of illness, thus relieving the approved societies or Insurance Committees of the necessity of making payments during this period of sickness, and in consideration of this undertaking to obtain for themselves and the nurses in their employ a reduction in the amount of the weekly contributions payable. When a nurse's right to sick pay is exhausted, because she has drawn it for twenty-six weeks, she is able, if still incapable of work, to draw 5s. a week so long as the incapacity continues—this is called disablement benefit. To become entitled to this benefit the nurse must have been insured for at least two years and must have paid at least 104 weekly contributions. Subject to this condition it will be possible for a nurse who is permanently incapacitated to be put into the receipt of an allowance of 5s. a week, but this allowance will cease, in the same way as sickness benefit, on the attainment of the age of seventy. At the age of seventy it is presumed the liability will be transferred to the National Old Age Pension Fund.

### Variation of Benefits on Marriage.

As most hospital nurses are unmarried the provision of maternity benefit does not seem at first sight to be of value. If, however, the nurse marries, and continues her hospital appointment or otherwise continues to be regularly employed, the maternity benefit will become payable on her confinement. The benefit is a payment of 30s., which, in the circumstances mentioned, she will receive in her own right as an employed contributor, and a further like sum will be payable to the husband if he, too, is an employed contributor under the Act. If upon her marriage she relinquishes regular employment she may elect to discontinue her contributions, or to continue at a special rate, receiving in return modified rates of benefit. Should she elect to discontinue her payments certain modified benefits may still be receivable. Her own and the hospital's previous contributions on her behalf will have enabled her society to set aside a certain sum for her benefit during marriage, and thus to pay her, on confinement, 5s. a week for not more than four weeks on any one occasion.

### Alternative Benefits.

It is possible that hospital nurses may be able to take advantage of Section 13 of the Act, by virtue of which power is conferred on the Insurance Commissioners to vary the benefits in certain cases. This variation in the benefits can only be obtained through an approved society; it is not possible for the benefits to be varied in the case of deposit contributors. The standard benefits, for which others may be given in substitution, are sickness and disablement benefits. A definite scheme must be submitted by the particular approved society, but it need not apply to all the members. It may apply to the whole of the members, or to any specified class of members, or it may be only for such members who elect to avail themselves of the substitution. In any case it will have to be shown that there are good reasons for desiring the substitution, and such benefits as are substituted will have to be shown to be equivalent in value to the sickness and disablement allowances relinquished. The substituted benefit or benefits must be chosen from among the list of additional benefits as enumerated in the Fourth Schedule to the Act. Amongst these the most suitable form of alternative benefit is the provision of pensions or superannuation allowances, or of the payment of contributions towards superannuation funds in which the members are interested. In those cases in which the hospitals pay their nurses either their full or a modified allowance whilst they are laid aside by illness, it would seem that very good reasons could be adduced whereby the special provision of this section could be invoked. It must not be forgotten that the scheme can only apply if application is made by an approved society. If the society does not take the initiative in the matter neither the employer nor the insured member can force it to do so. Nurses will, therefore, do well to join a society in which their interests are paramount, and in which by virtue of their membership they will have a controlling voice. For this reason, as well as the one we mentioned last week, nurses will consult their own best interests by joining the Nurses' Insurance Society.

### A Point for Hospital Managers.

There is one further section of the Act bearing on the insurance of nurses to which we must briefly refer. It is provided in Section 51 that "where the managers of any institution carried on for *charitable* or reformatory purposes prove that the persons who are *inmates* of and supported by the institution receive maintenance and medical attendance when sick, the Insurance Commisioners may grant a certificate of exemption to those managers, and where such a certificate of exemption is granted, any such inmates who are employed by the managers of the institution shall not, in respect of such employment, be deemed to be employed within the meaning of the Act." Such employed persons would not, therefore, be required to be compulsorily insured. Two words here require consideration. The first is the word "charitable," and it depends upon the decision as to whether or not a voluntary hospital comes within the meaning of that term as to the effect of the section on the insurance of hospital nurses. Advantage can only be taken of the section at the instance of the managers. The second word is "inmates"—and it is thought that this is sufficiently comprehensive to include the nursing staff. There are two important conditions which have to be satisfied by the managers before they can adopt the provisions of the section, and the effect of both of them is that on leaving the service of the institution the "inmate" shall not be in any worse position in regard to the insurance than would have been the case if the section had not been adopted. Thus it becomes necessary for the managers to pay in respect of any person who has been an inmate for at least six months, and who leaves the institution, such capital sum as will be sufficient to secure the statutory benefits at the full rate in the case of a person who was at the time of entering the institution below the age of sixteen. When the person was more than sixteen years of age on entering the institution, and was at that time an insured member of an approved society the payment to be made is a sum equal to the value of the contributions which would have been payable in respect of him during the time he or she was in the institution. These conditions appear to divest the section of any real advantage to the managers, and in consequence it would seem that in only exceptional cases will it be of importance.

---

### Answers to Correspondents.

### THE INSURANCE OF PUPILS.

"Enquirer" writes: "Will you kindly tell me in your valuable Insurance Article how the insurance will be paid in the following case?—County Nursing Associations send pupils for midwifery and district training to a training hospital for one year, paying all fees and expenses. Will the 6d. insurance have to be paid by the County Nursing Associations or by the training hospital? The pupils receive no wages."

⁂ It would seem that the pupils are not employed persons within the meaning of the Act, and in consequence they will not have to be insured until such time as their training is completed.—N.B.—Questions on all doubtful points are invited.

